# Effect of after‐school physical activity on body composition in primary school children: The Slovak “PAD” project

**DOI:** 10.14814/phy2.15540

**Published:** 2023-01-03

**Authors:** Roman Alberty, Ivan Čillík

**Affiliations:** ^1^ Department of Biology and Ecology Faculty of Natural Sciences of Matej Bel University Banská Bystrica Slovakia; ^2^ Department of Physical Education and Sports Faculty of Arts of Matej Bel University Banská Bystrica Slovakia

**Keywords:** BMI, body fat, physical intervention, schoolchildren

## Abstract

Physical activity is associated with many physical and mental health benefits. This study aimed to investigate the effect of a 24‐month after‐school physical activity intervention on body composition in normal‐weight children. Participating students (6–7 years of age at baseline) were divided by reason of their parental preference to intervention and control groups. Children in the intervention group (*n* = 20; 10 boys and 10 girls) followed an aerobic training program (two 60‐min sessions per week), whereas children in the control group (*n* = 20; 10 boys and 10 girls) participated in the usual practice. Body composition characteristics were repeatedly measured by means of bioelectrical impedance method. At 2 years, finally, intervention boys had a smaller rise in BMI (mean difference, MD: −0.97 kg/m^2^, *p* < 0.05), BMI *z*‐score (−0.44, *p* < 0.09), body fat % (BF%) (−6.47%, *p* < 0.01), and fat mass index (FMI) (−1.32 kg/m^2^, *p* < 0.001) than controls. In girls, however, the intervention program induced no significant differences (*p* > 0.9) in the measured variables compared to controls at the final follow‐up (MD: −0.04 kg/m^2^ for BMI and −0.01 for BMI *z*‐score). Changes in BF% and FMI in a positive direction occurred at 18 months (MD: −3.38%, *p* < 0.05 and −0.99 kg/m^2^, *p* < 0.01, respectively), but did not persist over time (*p* > 0.07). In addition, no significant changes (*p* > 0.07) in the fat‐free mass index were associated with the physical activity intervention in either boys or girls. In conclusion, compared to the controls, a long‐term physical activity intervention in boys was associated with a significantly smaller rise in BMI and improvement of body composition by reducing both BF % and FMI. In girls, however, this intervention did not result in any statistically significant changes in body composition variables.

## Graphical abstract

The study evaluated the long‐term effect of after‐school physical activity intervention on body composition in normal‐weight primary schoolchildren. Intervention boys showed a significantly smaller rise in BMI and lower values of body fat measures than controls. In girls, in contrast, enhanced physical activity had a marginally positive effect on BMI and body composition. Possible reasons for these gender‐specific differences are discussed.

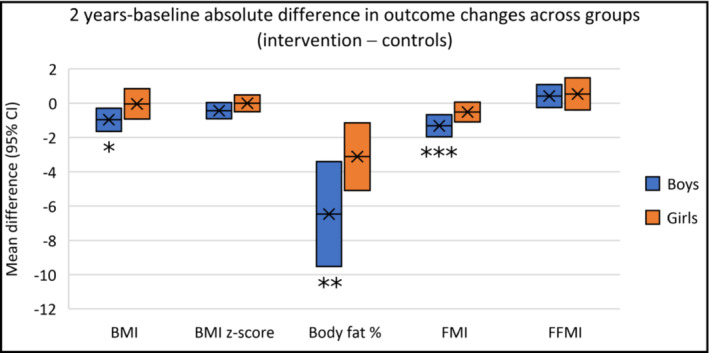

## 1 INTRODUCTION

Physical activity has many physical and mental health benefits during childhood and adolescence, including maintenance of energy balance and consequently healthy body weight, improvement of the cardiovascular system and mineral bone density, and reduction of anxiety and symptoms of depression (Tambalis, 2022). These benefits remain unchanged in children of all body mass index (BMI) categories (e.g., normal, overweight, and obese), and are also independent of age, gender, and health status (Hong et al., 2016). In contrast, physical inactivity leads to energy imbalance and childhood obesity and is associated with increased risk for the development of chronic diseases such as heart disease, diabetes mellitus, and some cancers (Blair, 2009).

The World Health Organization (WHO) recommends that children and youth aged 5–17 spend an average of at least 60 min per day in moderate to vigorous physical activity (MVPA) (WHO, 2020). According to the Health Behaviour in School‐aged Children (HBSC) survey, since 2014, levels of MVPA have declined in young people aged 11–15 years in roughly one third of European countries (Inchley et al., 2020). However, there is wide variance among countries. The lowest proportion of children meeting the recommended 60 min of MVPA was observed in Italy (13%), Denmark (15%), and Greece (16%), whereas the highest prevalence was found in Finland (41%), Ireland (38%), and Bulgaria (36%) (WHO, 2015). Survey data from the HBSC study revealed that only 28% of boys and 26% of girls at age 11 living in Slovakia achieved the recommended amount of physical activity based on the WHO guidelines; moreover, its level appeared to decrease with age in adolescence, with girls being less active than boys (Kopáčková, 2019).

Controlled school‐based physical activity interventions can increase the amount and intensity of physical activity in children (Kriemler et al., 2011). If changes in level of physical activity affect energy balance, this should result in changes in body weight or body composition. Recently Nikolic (2018) reviewed body composition changes in 20 closely related studies, which mostly employed the bioelectrical impedance method or dual‐energy x‐ray absorptiometry to measure changes in body composition in children and youth during an exercise intervention program. In all of them, except one study with a mind–body exercise, the authors concluded that physical activity has statistically significant positive effects on the body composition of children. The decrease in body fat mass in these studies varied between 2% and 24%. However, body fat loss may be lower in short‐term intervention studies (less than 6 months), in slim cohorts and in children younger than 10 years.

The purpose of this study was to evaluate the effect of a long‐term after‐school physical activity on body composition in 6‐ to 7‐year‐old children with normal body weight. We hypothesized that children participating in controlled physical activity intervention for 24 months would show a significant difference in BMI and other body fat indices compared to the control group.

## 2 METHODS

The “PAD” project (the Slovak acronym for *Pohybová Aktivita Detí*, i.e., “Children's Movement Activity”) was conducted in 2019 as a regional, population‐based survey that monitors the physical activity, health, and fitness levels of primary school‐age children in Banská Bystrica (a city with a population of 80,000) and its rural surroundings in Central Slovakia. The project combines questionnaire data, physical examinations, and a long‐term physical activity intervention program. The present study is based on the interventional part of this project and has a quasi‐experimental design.

### 2.1 Participants

A group of 40 healthy children of Caucasian origin was selected from Grade 1 of several classes at one city primary school to participate in a 24‐month intervention program. Suitable children met the following criteria: (1) they were 6–7 years old, (2) they were healthy and not suffering from eating disorders, (3) they regularly attended physical education lessons at school, (4) they did not participate in other organized sports activities in their free time, and (5) the children's legal guardians agreed to their participation in the project. On the request of their parents, 20 children (10 boys and 10 girls) were included in the intervention group; they followed a structured aerobic training program. The remaining children (10 boys and 10 girls) were assigned as controls; they were limited to regular physical education lessons without any intervention. The local Matej Bel University Ethics Committee approved the research protocol (ref. no. 4383/2019), which is consistent with the principles of the Declaration of Helsinki of 2000. All parents of participating children gave their written informed consent.

### 2.2 Intervention program

The 24‐month intervention program (twice a week, 60 min each time) was carried out on school premises under the supervision of a trained educator after lessons had finished. The training sessions drew on the national athletics program under the government‐sponsored IAAF Kids' Athletics project (International Association of Athletics Federations, 2006). The program was implemented with the aim of increasing primary school children's physical fitness based on regular athletics training, and of contributing to the comprehensive development of their physical abilities (coordination, balance, flexibility, muscular strength, and endurance). In regard to middle childhood, boys and girls participated in physical activity intervention on an equal basis, and most exercises were planned to involve at least 40 min of MVPA per sessions.

### 2.3 Anthropometric measures

An identical complete set of measurements was taken before the start of the study and subsequently at 6 months intervals for the duration of the study. Height was measured with electronic height rod (Soehnle). Weight and body composition (fat mass and body fat percentage, BF%) were determined with the child in light indoor clothing using InBody 120 (Biospace) bioelectrical impedance analyzer. The body composition analysis was performed on school premises at 10.00 a.m., before lunch, to ensure that the data were collected more than 3 h after waking up, and to avoid children eating and drinking at least 2 h prior to measurement. Prior data collection, the children were asked to urinate to follow standard procedure for bioelectrical impedance analysis.

### 2.4 Calculations and statistical analysis

BMI was calculated by the formula: BMI = [weight (kg) ÷ height (m)^2^]. The BMI *z*‐scores were derived from the Centre for Disease Control and Prevention (CDC) norm values for BMI (Kuczmarski & Flegal, 2000). Cut‐off points for overweight and obesity were based on the BMI‐related weight status categories for children aged 5–19 years (Cole et al., 2000). Fat‐free mass was calculated by the formula: fat‐free mass (kg) = weight (kg) × [1 – (BF% ÷ 100)] (Kouri et al., 1995). Fat mass index (FMI) was calculated as FMI = [fat mass (kg) ÷ height (m)^2^] and fat‐free mass index (FFMI) by the formula: FFMI = [fat‐free mass (kg) ÷ height (m)^2^] as described previously (VanItallie et al., 1990).

All statistical analyses were completed using the IBM SPSS software, version 28.0 (IBM Corp.). The Shapiro*–*Wilk test was used to ensure normality for data in each examined group. Continual outcomes are presented as mean and 95% confidence interval (CI) or median, and interquartile range (IQR). A mean difference (MD) measures the absolute difference between the mean value in the intervention group and control group. Categorical outcomes are described in absolute or relative frequencies (%). Fisher's exact test was used to compare input differences between categorical outcomes, while Student's *t*‐test for independent samples was used to examine the differences between the means of the two groups at the beginning of the experiment. A mixed‐model repeated measures ANOVA or the Friedman test were used to evaluate differences in intra‐ and inter‐group body composition, depending on the application conditions. The post hoc Bonferroni paired test or the Wilcoxon test and Bonferroni correction were used to evaluate significant differences in the pairwise comparison. Cohen's *d* effect sizes were calculated to indicate the standardized difference between the means of the intervention and control groups. The results are presented separately for boys and girls for different body composition values for each gender. Differences at *p* < 0.05 (one‐tailed) were considered statistically significant.

## 3 RESULTS

### 3.1 Participants characteristics and follow‐up.

The main demographic and anthropometric characteristics of the intervention and control groups at baseline level are listed in Table 1. There was no significant difference in mean age between the intervention and control groups. All the children in the intervention group were of normal weight; 2 children (10%, girls) in the control group were classified as having overweight. Baseline height, weight, and body composition characteristics in intervention and control groups were similar (*p* > 0.07).

**TABLE 1:** Baseline demographic and anthropometric characteristics for the intervention and control groups

Characteristics	Intervention group (*n* = 20)	Control group (*n* = 20)	*p*‐value^a^
Age (years)	6.9 (0.4)	6.8 (0.3)	0.404
Gender (M/F)	10/10	10/10	
Overweight (%)	0	2 (10)	0.244
Height (cm)	124.5 (4.6)	124.1 (2.9)	0.743
Weight (kg)	23.6 (2.8)	24.0 (2.7)	0.609
Waist‐to‐hip ratio	0.72 (0.03)	0.71 (0.03)	0.693
BMI (kg/m^2^)	15.23 (1.32)	15.71 (1.81)	0.347
BMI *z*‐score	−0.42 (1.10)	−0.09 (0.97)	0.165
Body fat (%)	14.83 (4.60)	17.37 (4.19)	0.075
Fat mass (kg)	3.7 (1.6)	4.6 (2.4)	0.163
Muscle mass (kg)	10.1 (1.3)	9.6 (0.7)	0.165

*Note*: Data are presented as means (SD) or frequencies (%).

Abbreviations: BMI, body mass index; F, female; M, male.

^a^ Student's *t*‐test for independent samples and Fisher's exact test.

The children included in the intervention group completed the full training program and all the children, regardless of the group, had all their body measurements taken. The participation of children from the intervention group in physical activities, defined as 120 min' participation per week, ranged between 75% and 90% over the 24‐month period.

### 3.2 BMI and body composition.

The development of anthropometric outcomes in boys in the intervention and control groups during the study period is shown in Table 2.

**TABLE 2:** Development of anthropometric outcomes in boys for intervention and control groups

Outcome		Mean					*p‐*value*	Mean difference in outcome changes across groups			
		95% CI						95% CI			
		Baseline	First half‐year	Second half‐year	Third half‐year	Fourth half‐year		1. Half‐year— baseline difference	2. Half‐year— baseline difference	3. Half‐year— baseline difference	4. Half‐year— baseline difference
BMI (kg/m^2^)	I	14.80 (13.88; 15.72)	14.60 (13.94; 15.27)	14.83 (14.09; 15.57)	15.23 (14–43; 16.03)	15.42 (14.71; 16.12)	0.008	−1.03 (−1.48; −0.58)	−1.36 (−1.97; −0.75)	−1.10 (−2.15; −0.05)	−0.97 (−1.64; −0.30)
	C	15.25 (14.59; 15.91)	16.09 (15.17; 16.99)	16.64 (15.56; 17.72)	16.78 (15.53; 18.03)	16.84 (15.77; 17.91)	<0.001	*p* ^#^ < 0.001	*p* = 0.003	*p* = 0.045	*p* = 0.048
BMI *z*‐score^a^	I	−0.84 (−1.39; 0.38)	−0.87 (−1.49; 0.04)	−0.65 (−1.68; −0.06)	−0.56 (−1.30; 0.23)	−0.56 (−1.24; −0.04)	0.235	−0.22 (−0.44, 0.01)	−0.42 (−0.85; 0.02)	−0.45 (−0.94; 0.04)	−0.44 (−0.92; 0.04)
	C	−0.06 (−1.17; 0.32)	0.34 (−0.55; 0.63)	0.54 (−0.12; 0.94)	0.31 (−0.26; 0.84)	0.33 (−0.10; 0.91)	0.002	*p* = 0.058	*p* = 0.073	*p* = 0.092	*p* = 0.089
Body fat, %	I	12.80 (9.90; 15.70)	11.48 (8.95; 14.01)	10.27 (7.78; 12.76)	9.80 (7.45; 12.15)	9.83 (7.66; 12.00)	<0.001	−0.94 (−1.55; −0.33)	−5.08 (−6.98; −3.18)	−6.03 (−9.23; −2.83)	−6.47 (−9.53; −3.41)
	C	15.99 (13.38; 18.60)	16.71 (14.06; 19.36)	18.54 (15.31; 21.77)	19.02 (15.15; 22.90)	19.49 (15.50; 23.48)	0.09	*p* < 0.001	*p* < 0.001	*p* = 0.004	*p* = 0.002
Fat mass index (kg/m^2^)	I	1.99 (1.50; 2.49)	1.68 (1.19; 2.17)	1.58 (1.10; 2.08)	1.64 (1.18; 2.09)	1.72 (1.22; 2.22)	<0.01	−0.62 (−0.82; −0.42)	−1.07 (−1.46; −0.68)	−1.15 (−1.81; −0.49)	−1.32 (−1.96; −0.68)
	C	2.45 (1.95; 2.96)	2.76 (2.19; 3.33)	3.12 (2.40; 3.85)	3.25 (2.39; 4.12)	3.50 (2.55; 4.45)	0.02	*p* < 0.001	*p* < 0.001	*p* < 0.001	*p* < 0.001
Fat‐free mass index (kg/m^2^)	I	12.66 (12.08; 13.24)	12.92 (12.41; 13.43)	13.22 (12.80; 13.63)	13.59 (13.05; 14.14)	13.63 (13.02; 14.24)	0.002	−0.27 (−0.46; −0.08)	−0.31 (−0.78; 0.15)	0.25 (−0.44; 0.94)	0.42 (−0.25; 1.09)
	C	12.75 (12.28; 13.22)	13.27 (12.79; 13.76)	13.62 (13.10; 14.13)	13.43 (12.96; 13.91)	13.30 (12.73; 13.87)	<0.001	*p* = 0.21	*p* = 0.22	*p* = 0.07	*p* = 0.07

*Note*: *p‐*value*, ANOVA‐repeated measures within‐group significance; *p*‐value^#^, ANOVA‐repeated measures between‐group significance (or Friedman test, if appropriate).

Abbreviations: BMI, body mass index; C, control group; CI, confidence interval; I, intervention group.

^a^ BMI *z*‐score is presented as median (interquartile range).

Over the course of the study, BMI increased significantly (*p* < 0.001) in both groups, except for the period of up to 1 year in the intervention group. The rise of BMI was smaller in the intervention group compared to the controls: the MD in BMI between the groups was −1.36 kg/m^2^ (95% CI: −1.97; −0.75, *p* < 0.01) in year 1 and − 0.97 kg/m^2^ (95% CI: −1.64; −0.30, *p* < 0.05) in year 2.

At the beginning, the boys in the intervention group weighed slightly less and were slightly taller than the boys in the control group (*p* > 0.6). The median BMI *z*‐score was lower in the intervention group −0.84 (IQR: −1.39; 0.38) than in the controls −0.06 (IQR: −1.17; 0.32). The negligible decrease (*p* < 0.10) in the BMI *z*‐score between the groups was similar in years 1 and 2, with MDs of −0.42 (95% CI: −0.85; 0.02) and − 0.44 (95% CI: −0.92; 0.04), respectively.

The BF% and FMI kept decreasing significantly (*p* < 0.01) in the intervention group; in the control group, there was a marginal increase in the BF% (*p* < 0.09) and a significant increase in the FMI (*p* < 0.02). Therefore, the MD in the BF% was −5.08% (95% CI: −6.98; −3.18, *p* < 0.001) in year 1 and −6.47% (95% CI: −9.53; −3.41, *p* < 0.01) in year 2; in the FMI it was −1.07 kg/m^2^ (95% CI: −1.46; −0.68) in year 1 and −1.32 kg/m^2^ (95% CI: −1.96; −0.68, both *p* < 0.001) in year 2 compared to the control group.

During the study, the FFMI increased significantly (*p* < 0.001) in both groups, though there was no significant difference between them: the MD in the FFMI was −0.31 kg/m^2^ (95% CI: −0.78; 0.15) in year 1 and 0.42 kg/m^2^ (95% CI: −0.25; 1.09, both *p* > 0.07) in year 2.

The development of anthropometric outcomes among the girls in the intervention and control groups over the course of the study is shown in Table 3.

**TABLE 3:** Development of anthropometric outcomes in girls for intervention and control groups

Outcome		Mean					*p*‐value*	Mean difference in outcome changes across groups			
		95% CI						95% CI			
		Baseline	First half‐year	Second half‐year	Third half‐year	Fourth half‐year		1. Half year—baseline difference	2. Half‐year—baseline difference	3. Half‐year—baseline difference	4. Half‐year—baseline difference
BMI (kg/m^2^)	I	15.67 (14.76; 16.57)	15.83 (14.89; 16.77)	16.15 (15.31; 16.99)	16.43 (15.53; 17.33)	16.43 (15.52; 17.35)	0.056	−0.30 (−0.76; 0.16)	−0.28 (−1.10; 0.54)	−0.19 (−1.01; 0.63)	−0.04 (−0.93; 0.85)
	C	16.17 (14.47; 17.87)	16.63 (14.95; 18.31)	16.94 (15.37; 18.50)	17.13 (15.27; 18.99)	16.98 (15.41; 18.55)	0.028	*p* ^#^ = 0.184	*p* = 0.543	*p* = 0.658	*p* = 0.939
BMI *z*‐score^a^	I	0.01 (−0.50; 0.82)	−0.10 (−0.39; 0.66)	−0.02 (−0.36; 0.59)	0.14 (−0.34; 0.71)	−0.06 (−0.29; 0.64)	0.735	−0.13 (−0.34; 0.08)	−0.21 (−0.60; 0.17)	−0.14 (−0.56; 0.28)	−0.01 (−0.50; 0.48)
	C	−0.02 (−0.60; 0.88)	0.13 (−0.27; 1.24)	0.34 (−0.39; 1.53)	−0.03 (−0.33; 1.43)	−0.13 (−0.62; 1.17)	0.049	*p* = 0.635	*p* = 0.933	*p* = 1.000	*p* = 1.000
Body fat (%)	I	16.85 (13.72; 19.98)	15.07 (12.13; 18.00)	14.83 (11.61; 18.01)	14.80 (11.75; 17.85)	14.81 (11.69; 17.93)	0.004	−2.35 (−3.34; −1.36)	−3.45 (−5.09; −1.81)	−3.38 (−4.92; −1.84)	−3.12 (−5.09; −1.15)
	C	18.75 (15.58; 21.92)	19.32 (16.47; 22.17)	20.18 (17.14; 23.22)	20.08 (17.15; 23.00)	19.83 (16.37; 23.30)	0.412	*p* = 0.002	*p* = 0.006	*p* = 0.013	*p* = 0.072
Fat mass index (kg/m^2^)	I	2.80 (2.00; 3.61)	2.52 (1.88; 3.17)	2.47 (1.79; 3.14)	2.31 (1.68; 2.94)	2.34 (1.67; 3.02)	0.065	−0.48 (−0.76; −0.20)	−0.97 (−1.57; −0.38)	−0.99 (−1.60; −0.38)	−0.52 (−1.10; 0.06)
	C	3.57 (2.13; 5.00)	3.77 (2.38; 5.16)	4.20 (2.77; 5.63)	4.06 (2.86; 5.27)	3.63 (2.49; 4.78)	0.156	*p* = 0.014	*p* = 0.007	*p* = 0.010	*p* = 0.250
Fat‐free mass index (kg/m^2^)	I	12.89 (12.45; 13.34)	13.33 (12.86; 13.80)	13.69 (13.28; 14.09)	14.05 (13.53; 14.56)	14.09 (13.54; 14.64)	0.002	0.00 (−0.20; 0.20)	−0.12 (−0.46; 0.22)	0.62 (−0.08; 1.32)	0.54 (−0.40; 1.48)
	C	12.44 (12.02; 12.86)	12.87 (12.45; 13.29)	13.35 (12.84; 13.87)	12.97 (12.24; 13.70)	13.09 (12.04; 14.14)	0.010	*p* = 0.953	*p* = 0.656	*p* = 0.103	*p* = 0.230

*Note*: *p‐*value*, ANOVA‐repeated measures within‐group significance; *p*‐value^#^, ANOVA‐repeated measures between‐group significance (or Friedman test, if appropriate).

Abbreviations: BMI, body mass index; C, control group; CI, confidence interval; I, intervention group.

^a^ BMI *z*‐score is presented as median (interquartile range).

The BMI increased significantly over time in the control group (*p* < 0.05); the BMI increase in the intervention group was marginally significant (*p* < 0.06). The intergroup MD in BMI was −0.28 (95% CI: −1.10; 0.54) in year 1 and −0.04 (95% CI: −0.93; 0.85, both *p* > 0.18) in year 2.

At the beginning, the median BMI *z*‐score was 0.01 (IQR: −0.50; 0.82) in the intervention group and −0.02 (IQR: −0.60; 0.88) in the control group. The intergroup MD in the BMI z‐score increased negligibly (*p* > 0.6) during the study, from −0.21 in year 1 to −0.01 in year 2.

The positive trend observed after 18 months between groups for the BF% ‐3.38 (95% CI: −4.92; −1.84, *p* < 0.05) and for the FMI ‐0.99 kg/m^2^ (95% CI: −1.60; − 0.38, *p* < 0.01), did not subsequently persist (*p* > 0.07).

**FIGURE 1:**
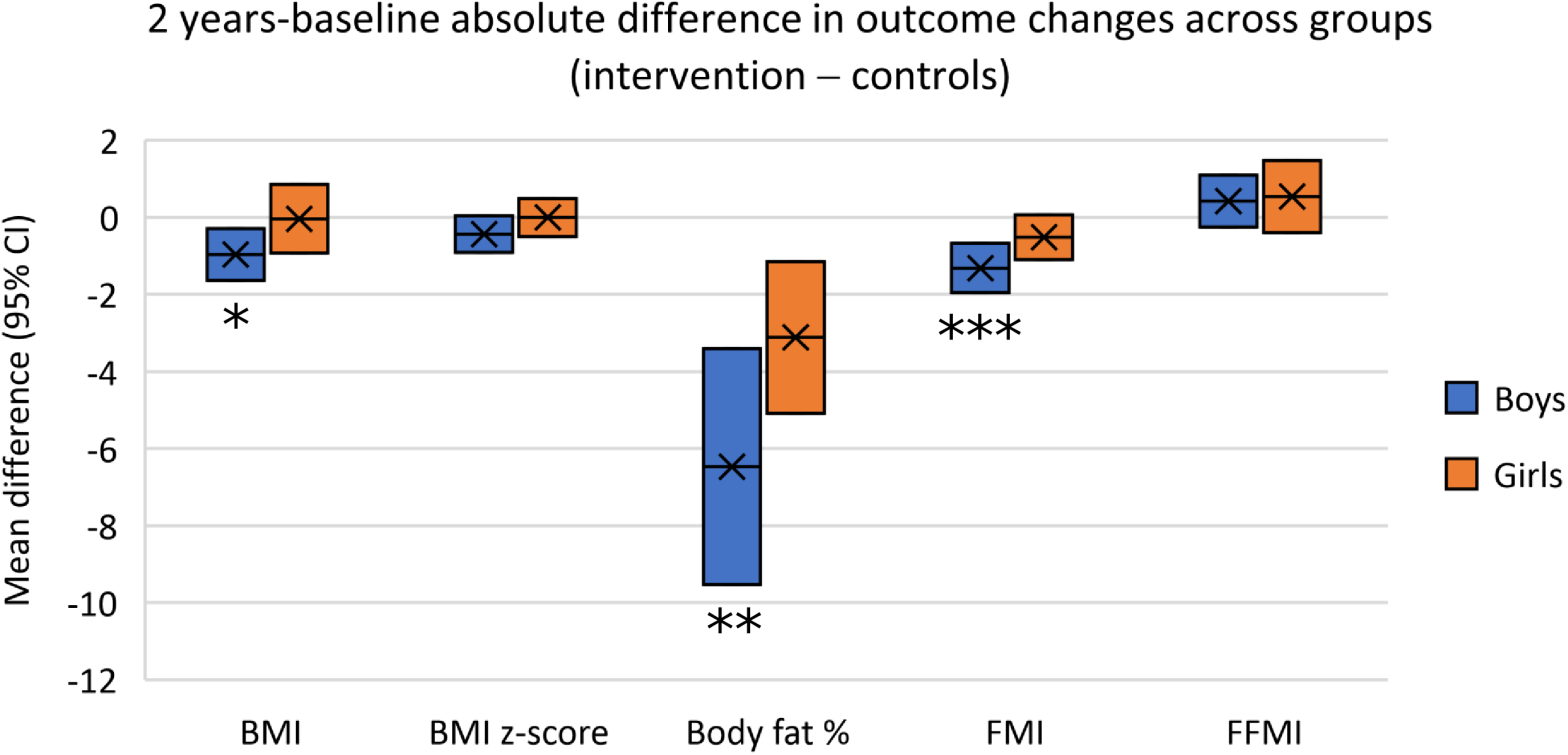
Two years–baseline absolute mean differences (95% CI) in body composition changes across groups (intervention – controls). The *p*‐value is given for intervention effect: *p < 0.05, **p < 0.01, ***p < 0.001. BMI, body mass index; CI, confidence interval; FFMI, fat‐free mass index; FMI, fat mass index.

As with the boys, over the course of the study the FFMI increased significantly (*p* < 0.001) among girls in both intervention and control groups, but with no significant differences between them, that is, −0.12 (95% CI: −0.46; 0.22) in year 1 and 0.54 (95% CI: −0.40; 1.48, both *p* > 0.2) in year 2. In general, a 2‐year physical activity intervention for girls is not associated with significant changes in BMI and body fat composition as in boys (Figure 1).

## 4 DISCUSSION

The aim of the presented study was to investigate the effect of after‐school physical activity intervention on BMI and body composition in younger normal‐weight school‐age children. Our findings showed that the 2‐year intervention program resulted, among boys, in a smaller rise of BMI and improved body composition by reducing both BF % and FMI. Nevertheless, physical activity intervention had no positive, or only a marginal, effect on the BMI and body composition of young girls.

Physiologically, body composition is the proportion of body fat and non‐fat mass. It changes dramatically over the lifespan (chronological age) in humans (Ryan & Elahi, 2007). These changes in particular need to be carefully considered in the pediatric population, where body composition reflects the growth spurt and puberty in both sexes (Wells, 2003). Body composition is largely regulated by endocrine factors, with critical roles played by growth hormone and gonadal steroids (Loomba‐Albrecht & Styne, 2009). Several cross‐sectional observations in children and adolescents indicate that BMI and body fat naturally increase with age, moreover, in all possible weight categories that is, from permanent normal weight to permanent obesity (Cole et al., 2000; Lin et al., 2014). The timing of those changes is thought to be genetically regulated throughout life (Sovio et al., 2011).

Most studies examining the effect of physical activity intervention on body composition concentrate on overweight and obese school‐age children (Mead et al., 2017). Furthermore, intervention programs usually have several components: physical, dietary, and behavioral (Serra‐Paya et al., 2015). Long‐term studies (6 months or longer) evaluating solely effect of after‐school physical activity on young schoolchildren of healthy weight are scarce and have shown inconsistent results in terms of body composition. In this context, three studies designed along lines similar to ours (a quasi‐experimental study, only the impact of after‐school physical activity under the guidance of a trained educator, a long‐term intervention program, and a comparison with a control group) can serve as good examples.

In one study, 140 girls and 158 boys from Denmark aged 8–10 regularly attended sports clubs. Their BMI was 5% smaller and their FMI was 16% lower (*p* < 0.05) than children who were not active in sports clubs. Nevertheless, the difference in fat content was significant for girls (21%), but not for boys (6%), and was confirmed only for girls who engaged in ball games, but not girls doing other sports (Larsen et al., 2017). Another study, conducted among 118 African American girls aged 8–12 years, focused on the effect of a 10‐month intervention program (80 min per week, of which 35 min MVPA) on physical fitness and body composition, and described a reduction in their BMI by 2.9% and in their BF% by 2% (*p* < 0.001) compared to the control group (Barbeau et al., 2007). More recently, a 6‐month fitness intervention program for 14 Canadian schoolchildren aged 5–12, who engaged in structured physical activity at least 3 days a week with an MVPA intensity of 60 min per day, did not yield a significant improvement in body composition; their BMI decreased by 1.8% compared to the control group, and their BF% was reduced by 6.8% (*p* > 0.12) (Crozier et al., 2022).

In the current research, the children's BMI increased continuously over 24 months across groups, except for the first 12 months among boys in the intervention group. At 2 years, finally, boys who underwent the intervention showed a 6.2% (*p* < 0.05) smaller rise in BMI compared to their less active counterparts. The difference observed in BMI between these groups is greater than that has been reported in other studies (Larsen et al., 2017; Martínez‐Vizcaíno et al., 2008). In contrast, physical activity in the girls in the intervention group did not result in significant BMI changes (*p* > 0.2) over the study period compared to the control group; this is inconsistent with the findings described after the physical activity intervention among US black and Danish schoolgirls (Barbeau et al., 2007; Larsen et al., 2017). Nevertheless, BMI is a crude indicator and not accurately reflects changes in the two body components, fat mass and fat‐free mass, and therefore it is possible to observe different associations in relation to physical activity ad hoc (Reisberg et al., 2020).

The mean BMI *z*‐score of our girls at baseline fell close to the CDC median of 2000 charts (Cole et al., 2000); the mean BMI *z*‐score of the boys in the intervention group (−0.85) and control group (−0.30) were 26th and 42th percentiles, respectively, according to the same growth standards. Although the mean difference in the boys' BMI *z*‐score at the end of the intervention period was non‐significant (−0.44, *p* < 0.09), the potency of the physical activity effect size was strong (Cohen's *d* = 1.12). In contrast, in girls, the potency of the physical activity effect relative to the BMI *z*‐score was very small (*d* = 0.08) and only reflected small inter‐group differences in BMI (*z*‐score). However, for example, the results of the recent Italian study showed that the school‐based intervention program was associated with a significant favorable change for BMI *z*‐score in physically active girls in puberty even with a normal body weight (Ermetici et al., 2016).

All three studies designed along similar lines, as discussed above, also included a BF% determination. Two of them, contrary to our study, found significant differences in BF% between the intervention and control groups in girls following the long‐term intervention program (Barbeau et al., 2007; Larsen et al., 2017). And two of these three studies, which also examined boys, did not provide evidence that physical activity significantly affects the BF%; this also contradicts our own findings, which convincingly showed a positive trend in body composition, namely, a significant reduction in the BF% and FMI of boys during and after the intervention program (Crozier et al., 2022; Larsen et al., 2017). In our study, BMI correlated effectively with body fat. The linear relationship between BMI and BF% was shown to be greater among the girls (*R* ^2^ = 0.70) than the boys (*R* ^2^ = 0.52, both *p* < 0.001). This strong relationship has been reported in several studies (Costa‐Urrutia et al., 2019; Wang & Hui, 2015). The FMI used in our study is considered to be a more sensitive indicator of obesity than BF% or fat mass because it also reflects the child's height in the body composition (Demerath et al., 2006). Over the course of the study, intervention boys reported a significant decrease (*p* < 0.01) and intervention girls showed a marginally downtrend (*p* < 0.07) for BF% and FMI. The negative fat balance observed in our and similar intervention studies can probably be attributed to increased fat oxidation and the increased oxidative capacity of skeletal muscle fat during regular exercise (Jeukendrup, 2002). Our study also confirmed a higher BF% and FMI in less active children, as has been previously described in children and adolescents (Larsen et al., 2017; Simon et al., 2008).

For boys and girls in our study, the FFMI rise (*p* < 0.01) consistently during the intervention period. BMI is composed mainly of FFM, that is, FFMI is 5–8‐fold and 3–5‐fold greater than FMI for intervention and control groups, respectively. However, we did not register any positive effect of physical activity on the FFMI of children who participated in the intervention program, as was also the case in several studies (Larsen et al., 2017; Simon et al., 2008). Most of the training sessions in our program were focused on structured physical activities that are more aerobic in nature than resistant exercises for muscle hypertrophy, which could explain the lack of a significant inter‐group effect on the FFMI (*p* > 0.1). This reasoning is supported by a study comparing the effects that intense training among 10 female gymnasts aged 7–12 and the moderate exercise of their peers have on protein metabolism. There were no between group differences in protein intake, protein synthesis, or breakdown (Boisseau et al., 2005).

Small effect of after‐school physical activity intervention program on girls' BMI and body composition is an unexpected finding of this study. It is widely believed that girls may be more receptive to intervention programs that promotes weight control (Poitras et al., 2016). The reason why the intervention program was ineffective for improving of body composition in girls (with results often the opposite to those seen in boys), is not entirely clear. However, the specific characteristics of each intervention (e.g., length of the program, frequency of sessions, intensity of exercise or level of parental involvement) may be more effective in boys than girls, and vice versa. We can also consider other explanation that the unexpected results of the intervention programs are not always related to the physical activity itself, but to the moderators of this relationship or confounder variables (Bauman et al., 2002).

On the other hand, it has been well‐documented that exercise can modulate via epigenetic mechanisms the expression of several human skeletal muscle genes (Jacques et al., 2019). This opens up the scenario that lifestyle factors may interact with the developmental program involved in regulating the amount of body fat and its distribution in the body. Theoretically, compared to boys, genetic developmental factors may have much greater effect on growth and changes in body composition among girls at this age than other factors linked to physical activity.

In our study, physical exercises and the management of the intervention program were carried out by a trained educator to ensure that the trainings included WHO‐recommended MVPA. Similarly, parental indirect involvement was key to the success of the intervention program (social background, motivation, the taking of the children to sessions). The high participation in the intervention program can also be explained by the fact that the exercises were organized in a familiar school environment and after school. The importance of this program also lay in the fact that it was able to favorably modify the usual daily activities of the children. It is well known that a beneficial indirect effect of increasing physical activity in spare time is a reduction in the amount of time spent watching television, playing computer games, and the associated consumption of unhealthy food (Greier et al., 2019). The results of our study seem to be in line with current systematic reviews that even a relatively small increase of physical activity on a regular basis can have significant health effects in association with other changes to the daily routine (Demetriou et al., 2017; Verjans‐Janssen et al., 2018).

The present study has some strengths. First, the intervention was carefully planned and conducted to ensure the WHO‐recommended intensity of physical activity. Second, it covers the critical period when children are starting their schooling, which is a time when physical activity decreases and a sedentary lifestyle increases. Third, it showed that physical activity intervention in after‐school regime can significantly improve body composition even in children with normal weight.

Some limitations of this study must be also considered. First, the small sample size of examined children, which limits the ability to generalize the results obtained to a larger part of pediatric population. Second, a participation bias in that slim children and children from a motivating home environment were the ones showing most interest in physical activity intervention. Finally, we did not have detailed information on the full range and all forms of the children's everyday physical activity at school and in their leisure time. However, together all the children completed all the 90 minutes of compulsory school physical education per week.

## 5 CONCLUSION

This 2‐year controlled study indicates that the after‐school physical activity intervention oriented to normal‐weight primary school‐age children resulted in a significantly smaller rise in BMI and improved body fat composition in boys, but had no positive, or only marginal, effect on the BMI and body composition in girls. For younger girls, however, it is necessary to find a different, multi‐component strategy to improve their body composition. In general, the results of our study are in line with the recent observation that even a moderate amount of exercise in normal‐weight youths can serve as an effective approach in preventing of overweight and obesity. Further studies will be needed to evaluate the development of children's body composition variables in the period after the end of the intervention program.

## AUTHOR CONTRIBUTIONS

R.A. and I.Č. conceptualized the study and designed the experiment. I.Č. performed training procedures and collected data. R.A. analyzed the data and prepared draft of the paper. R.A. and I.Č. contributed significantly to the revision of the paper for intellectual content and reviewed the final draft.

## FUNDING INFORMATION

This study was supported with a financial grant from the Ministry of Education, Science, Research and Sport of the Slovak Republic (VEGA 1–0491‐20).

## CONFLICT OF INTEREST

The authors declare no competing interests.

## ETHICS STATEMENT

This study was approved by the Matej Bel University Ethics Committee, Slovakia (ref. no. 4383/2019). All parents of participating children provided informed written consent before participation.
